# Beam Tracking X-Ray Phase-Contrast Imaging Using a Conventional X-Ray Source

**DOI:** 10.3390/s25196089

**Published:** 2025-10-02

**Authors:** Jiaqi Li, Jianheng Huang, Xin Liu, Yaohu Lei, Botao Mai, Chenggong Zhang

**Affiliations:** 1 Key Laboratory of Optoelectronic Devices and Systems of Ministry of Education and Guangdong Province, College of Physics and Optoelectronic Engineering, Shenzhen University, Shenzhen 518060, China; ljq18811839677@163.com (J.L.); yfyt10@163.com (Y.L.); maitian628@gmail.com (B.M.); 2 Institute of Advanced Light Source Facilities, Shenzhen 518107, China; chenggong_zhang@163.com

**Keywords:** X-ray phase-contrast imaging, conventional X-ray source, pinhole array modulator

## Abstract

**Highlights:**

**What are the main findings?**

- An X-ray beam tracking phase-contrast imaging system based on a conventional X-ray source was experimentally demonstrated.
- The method maintains high sensitivity under large focal spot conditions and enables the extraction of phase gradient information.

**What is the implication of the main finding?**

- The system can be implemented under standard laboratory conditions.
- It shows promising potential for nondestructive imaging of low-density, weakly absorbing, or biological materials.

**Abstract:**

To address the issue of insufficient contrast in conventional X-ray absorption imaging for biological soft tissues and weakly absorbing materials, this paper proposes a beam tracking X-ray phase-contrast imaging system using a conventional X-ray source. A periodic pinhole array mask is placed between the X-ray source and the sample to spatially modulate the X-ray beam, dividing it into multiple independent sub-beams. Each sub-beam is deflected due to the modulation effect of the sample, resulting in slight positional shifts in the intensity patterns formed on the detector. The experiments employ an X-ray source with a 400 μm focal spot and use a two-dimensional step-scanning approach to acquire image sequences of various samples. The experimental results show that this method can extract the edge profile and structural changes in the samples to some extent, and it demonstrates good contrast and detail recovery under weak absorption conditions. These results suggest that this method has certain application potential in material inspection, non-destructive testing, and related fields.

## 1. Introduction

Since the discovery of X-rays by Wilhelm Röntgen in 1895, X-ray imaging technology has been widely applied in various fields such as medical diagnostics, industrial nondestructive testing, materials science, and biology due to its excellent penetration capability. Traditional X-ray imaging is primarily based on the absorption differences in materials, with image contrast depending on atomic number. However, elements with high atomic numbers exhibit strong absorption of X-rays, while those with low atomic numbers absorb weakly. As a result, X-ray absorption imaging is less effective for light-element materials such as biological soft tissues, polymers, and fiber-reinforced composites, greatly limiting its application in soft tissues and low-density materials. In contrast, light-element materials cause much larger phase shifts in X-rays than changes in amplitude [1]. X-ray phase-contrast imaging detects the phase variations in X-rays within an object, enabling the acquisition of richer structural details compared to absorption imaging [2]. Moreover, it is particularly suitable for light-element materials. In X-ray phase-contrast images, fine structures that are invisible in absorption images are often clearly observed. As a result, phase imaging is considered an important technique for enhancing image contrast and spatial resolution.

The most commonly used X-ray phase-contrast imaging techniques currently include interferometry [3], carrier-based modulation methods [4,5,6,7,8], free-space propagation methods [9,10], and beam tracking methods [11]. Among them, interferometry primarily utilizes the interference between a reference beam and an object beam to convert phase variations in the object beam into intensity variations. Its main applications include X-ray phase-contrast microscopy and crystal interferometer imaging. Carrier-based modulation methods leverage the modulation effects induced by the sample on the X-rays after transmission; by analyzing the intensity distributions before and after modulation, information on refraction and scattering within the sample can be extracted. Typical applications include diffraction-enhanced imaging [12], grating-based phase-contrast imaging [13,14,15], and speckle-based phase-contrast imaging [16]. Free-space propagation methods are a type of phase-contrast imaging technique that require no additional optical components and rely on the interference and diffraction effects generated during the free propagation of X-rays beyond the sample.

Beam tracking (BT) is a simple yet effective technique for extracting phase information without relying on interference, and it has developed rapidly in recent years. The origin of the BT method can be traced back to 2007 when A. Olivo et al. proposed the coded aperture X-ray phase contrast imaging technique [17]. They used a slit array as a mask at the sample end to split the beam from a conventional X-ray source into multiple micro-beams; simultaneously, another matching mask was placed in front of the detector so that the beams just illuminated the edges of the pixels. This method uses matched double masks to convert the small phase shifts caused by the sample into measurable intensity variations. Subsequently, C. Navarrete-León et al. extended this method to bidirectional Beam Tracking (2DBT) imaging, achieving the simultaneous acquisition of phase gradient information in two orthogonal directions [18]. However, it should be noted that they still used a micro-focus X-ray source, rather than a conventional large-focus source.

On the other hand, the concept of sampling X-rays using masks and analyzing beam modulation is also related to earlier spatial harmonic imaging (SHI) methods, which use two-dimensional grids for harmonic separation in the Fourier domain to obtain absorption and phase contrast information of the sample [19,20]. Another related concept is the Hartmann mask, which uses a regular aperture array to generate a localized beam; By monitoring the intensity, displacement, and broadening of these beams, absorption, refraction, and scattering information can be extracted [21,22]. Therefore, BT can be seen as a generalization of these earlier methods, providing a simple spatial domain implementation with low coherence requirements, making it ideal for traditional laboratory X-ray sources.

In 2015, Fabio A. Vittoria et al. implemented beam tracking phase-contrast imaging using a microfocus X-ray source, simultaneously extracting absorption, refraction, and scattering information from the sample in a single exposure [23]. In the study by Erik S. Dreier et al., they employed an absorption-mask-based beam tracking method to achieve single-exposure, two-dimensional dark-field imaging under laboratory conditions [24]. By analyzing the changes in intensity distribution of each micro-beam on the detector, they extracted scattering information caused by the internal structure of the sample.

Most early research on beam tracking and related technologies relied on micro-focused X-ray tubes or synchrotron radiation sources, where the focal size is typically limited to a few microns to several tens of microns. Combined with high-resolution detectors, this enables precise tracking of individual microbeams. Olivo et al. also demonstrated that phase contrast imaging using conventional larger focal source coding aperture methods is feasible, mainly serving as a proof of concept. In contrast, the current work extends the applicability of beam tracing to larger focal conditions (hundreds of micrometers) and implements a two-dimensional pinhole array structure based on Fourier subpixel displacement analysis, thereby allowing for the extraction of quantitative phase gradients under standard laboratory conditions.

## 2. Principles and Methods

**Figure 1:**
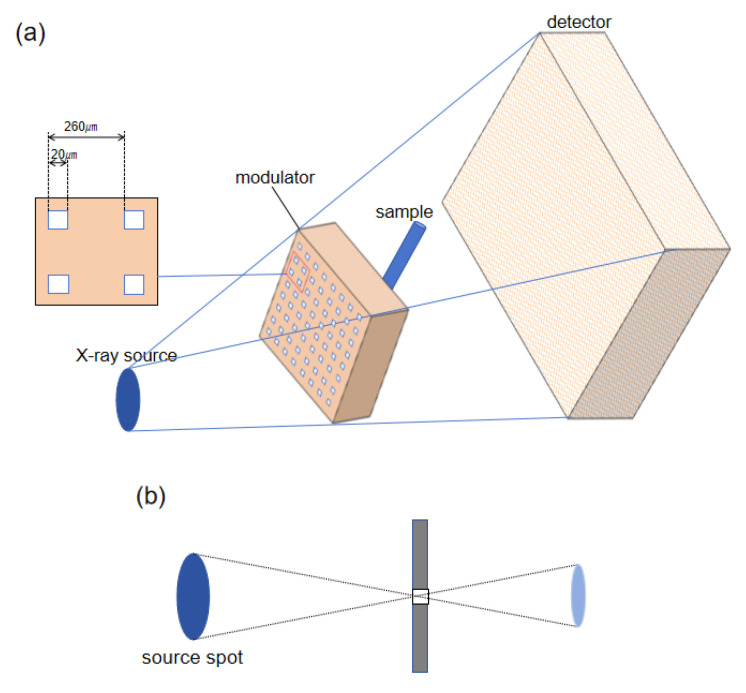
(**a**) Schematic diagram of the phase-contrast imaging system structure; (**b**) Schematic illustration of the Pinhole image pattern of the X-ray source spot.

The X-ray phase-contrast imaging system proposed in this study consists of three main components: an X-ray source, a pinhole array modulator, and a detector, as illustrated in Figure 1a. The core component of the system is the pinhole array modulator, which spatially modulates the light emitted from the source, dividing the continuous X-ray beam into multiple independent micro-beams. Variations in the sample’s refractive index cause slight deflections of the micro-beams, leading to subtle positional shifts on the detector and forming intensity distributions that reflect local structural features of the sample.

The pinhole array modulator used in the study consists of a series of periodically arranged square apertures designed to spatially modulate the continuous X-ray beam from the source, producing multiple spatially separated micro-beams after transmission. The aperture size of the modulator is 20 µm, with an inter-aperture spacing of 260 µm. It was fabricated from tungsten with a thickness of 100 μm.

After the X-ray micro-beams pass through the modulator and are modulated by the sample, their intensity distributions on the detector change accordingly due to absorption, refraction, and scattering effects. By extracting and analyzing the features of each micro-beam, one can separately reconstruct the sample’s absorption image and phase gradient image, thereby achieving comprehensive X-ray imaging.

**Figure 2:**
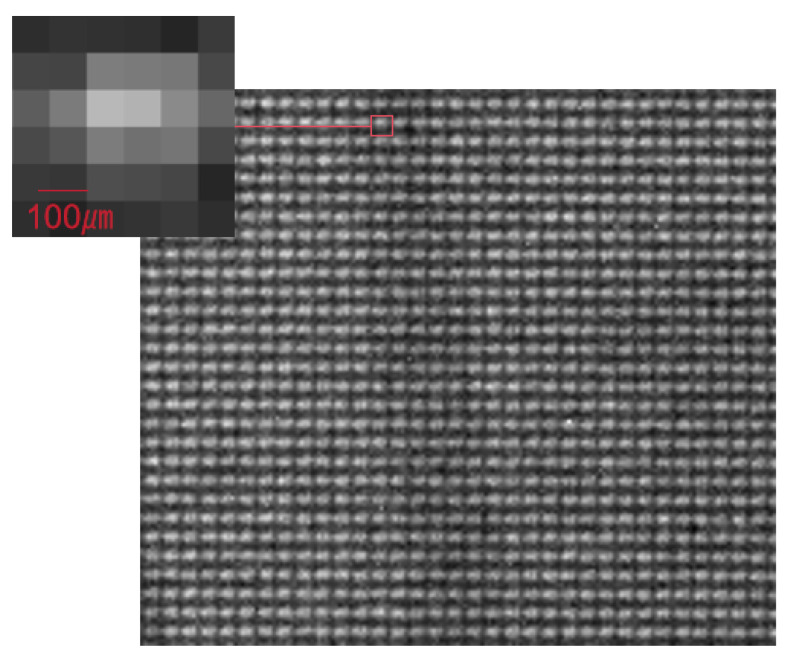
Image at actual pixel resolution: intensity distribution array of micro-beams captured on the detector.

Figure 2 shows an experimentally acquired image at the detector’s actual pixel resolution, where the intensity distribution array of the micro-beams on the detector can be clearly observed. This image corresponds to a single step in the two-dimensional step-scanning process. By translating the sample point by point using a motorized stage, images at different positions are captured, clearly revealing the periodic spatial distribution of the modulation array. The detector used has a pixel size of 74.8 µm. A zoomed-in view of a single spot in the modulation pattern is provided to illustrate the spatial intensity profile of an individual micro-beam at the detector pixel scale, which is then used to calculate the phase modulation introduced by the sample. From the image, it can be seen that although the X-ray source in this study has a relatively large spot size of several hundred micrometers, the mask structure still effectively modulates the beam, preserving distinguishable micro-beam features on the detector. In particular, the magnified local view clearly shows the intensity distribution of each micro-beam on the detector.

To extract the phase modulation information induced by the sample on the X-ray wavefront, we analyze the small displacements caused by the sample. When a uniform plane wave is emitted from the X-ray source, it is modulated by the periodic aperture structure of the mask into multiple quasi-parallel beams. As each beam passes through the sample, its propagation direction is slightly deflected due to local phase gradients. These displacement amounts, Δx and Δy, are proportional to the phase gradients ∂φ/∂x and ∂φ/∂y introduced by the sample.

Assume that the reference image recorded on the detector without the sample is denoted as f_r,m,n_(x,y); after the sample is inserted, due to refractive effects, the local intensity in the image undergoes sub-pixel displacement, resulting in a new image f_s,m,n_(x,y). This displacement can be described by the following expression:

$$f_{r,m,n}(x,y)=f_{s,m,n}(x-\Delta x,y-\Delta y),$$

where Δx and Δy represent the intensity displacement of each light spot caused by the sample’s refractive effect, while m and n denote the position indices of the light spots in the image.

To accurately extract these small displacements from the images, we employ the Fourier shift theorem for analysis. By transforming the above expression into the frequency domain, we obtain:

$$F_{r,m,n}(u,v)=F_{s,m,n}(u,v)e^{-i2\pi (u\Delta x+v\Delta y)},$$

where F_r,m,n_(u,v) and F_s,m,n_(u,v) are the two-dimensional Fourier transforms of f_r,m,n_(x,y) and f_s,m,n_(x,y), respectively, and u, v are the spatial frequency variables in the x and y directions.

Subsequently, we choose two spatial frequency points to calculate the displacement. In principle, any two frequency points can be selected. However, for convenience in calculations, it is common to take one component as zero, thus selecting (u, 0) and (0, v), and obtain:

$$F_{r,m,n}(u,0)=F_{s,m,n}(u,0)e^{-i2\pi \cdot u\Delta x}\;F_{r,m,n}(0,v)=F_{s,m,n}(0,v)e^{-i2\pi \cdot v\Delta y}.$$

By extracting the corresponding phase angles, the displacements can be expressed as:

$$\Delta x=-\frac{1}{2\pi u}\arg[\frac{F_{r,m,n}(u,0)}{F_{s,m,n}(u,0)}]\;,\;\;\Delta y=-\frac{1}{2\pi v}\arg[\frac{F_{r,m,n}(0,v)}{F_{s,m,n}(0,v)}],$$

where arg refers to the phase angle extracted from the complex Fourier coefficients.

Once the displacements Δx and Δy induced by the sample have been calculated, the phase gradients in the x and y directions can be determined accordingly [25].

$$\frac{\partial \varphi }{\partial x}=k\frac{\Delta x}{z}\;,\;\frac{\partial \varphi }{\partial y}=k\frac{\Delta y}{z},$$

where k = 2π/λ is the wave number, and z is the distance between the sample and the detector. Based on the known displacements, the phase gradients of the wavefront in the x and y directions can thus be calculated.

Furthermore, the phase variation induced by the sample on the wavefront can be reconstructed using the following expression.

$$\varphi (x,y)=\mathrm{FF}T^{-1}[\frac{FFT[\partial \varphi /\partial x+i\partial \varphi /\partial y](u,v)}{2\pi i(u+iv)}](x,y),$$

where φ(x,y) represents the two-dimensional phase distribution to be recovered; ∂φ/∂x and ∂φ/∂y denote the phase gradient images of the sample in the x and y directions, respectively. The variables u and v represent the spatial frequencies corresponding to the x and y directions in the frequency domain. Based on the principle of frequency-domain integration, the phase gradients in both directions can be combined in a complex form for integration, enabling efficient reconstruction of the original phase map φ(x,y).

## 3. Experiments and Discussion

To further verify the applicability of the proposed method in practical imaging scenarios, we built an experimental platform and conducted tests on multiple sample types. The X-ray source (Varian Medical Systems, Palo Alto, CA, USA; model HPX-160-11, anode material: tungsten) used in the experiment was operated at 40 kV tube voltage and 3 mA tube current, with an effective X-ray energy of about 25 keV and a nominal focal spot size of approximately 400 μm. The detector (Dexela, London, UK; model 2923M) was employed, and the exposure time was 2 s per frame. To obtain complete information about the sample, a two-dimensional step-scanning approach was employed during data acquisition, and a total of 13 × 13 image frames were obtained. This scanning strategy ensured sufficient spatial coverage to sample the entire structure of the specimen. During imaging, after the X-rays pass through the pinhole array, they are modulated by the sample, and the detector captures the resulting modulated intensity pattern. By comparing the background image sequence (without the sample) and the sample image sequence obtained through step scanning, the displacement of each micro-beam induced by the sample at different positions can be extracted. These displacements are then used to calculate the phase gradients and reconstruct the internal structural information of the sample.

In the experiment, a pencil, a polymethyl methacrylate(PMMA) rod, and a polyoxymethylene(POM) standard rod were selected as test samples for phase imaging to evaluate the method’s adaptability and sensitivity to different materials. These three samples each represent distinctive internal structures and interface characteristics, providing a comprehensive basis for evaluating the imaging performance of the system under various material conditions. In the experiment, the distance between the light source and the hole array modulator was 93 cm, and the distance between the hole array and the detector was 47 cm. It is worth noting that the sample placed behind the hole array should be kept at a distance of approximately 29 cm from the detector.

Specifically, the pencil consists of a high-density graphite core and a low-density wooden shell, featuring a distinct layered structure and sharp density transitions. PMMA, as a transparent polymer material, exhibits good homogeneity and is well-suited for evaluating the system’s response under conditions of weak absorption and small phase gradients. In contrast, the POM rod is a highly crystalline engineering plastic with relatively high density and strong refractive index gradients. Its well-defined interfaces and stable edges make it ideal for assessing the imaging system’s accuracy in responding to microstructural boundaries and edge features.

**Figure 3:**
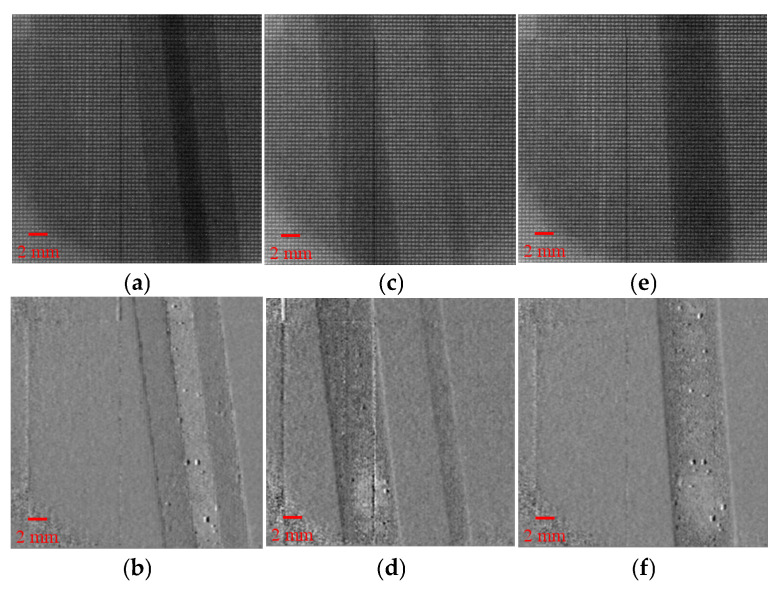
Phase gradient reconstruction results of three material samples. (**a**,**b**) Raw modulation image and reconstructed phase gradient image of the pencil sample; (**c**,**d**) Raw and phase gradient images of the PMMA rod; (**e**,**f**) Raw and phase gradient images of the POM rod.

Figure 3 presents the phase gradient reconstruction results for the three types of material samples obtained during the experiment. Figure 3a,b show the raw modulation image and the reconstructed phase gradient image of the pencil sample; Figure 3c,d correspond to the PMMA rod; and Figure 3e,f to the POM rod. As can be seen from the images, all samples exhibit clear edge contrast and detailed structural features in their reconstructed phase gradient images, indicating that the structural information has been effectively recovered through the reconstruction process. It is important to note that the vertical lines visible in Figure 3a,c,e are not caused by the reconstruction method, but by defective pixels in the detector.

Taking the pencil sample as an example, the phase image clearly reveals the density difference between the inner graphite core and the outer wooden shell. Notably, the image maintains good contrast and continuity, especially at the edges and in the interlayer details.

Although the PMMA sample has a uniform internal structure and weak absorption characteristics, its edge contours are still discernible in the phase gradient image, demonstrating the high sensitivity of the proposed method for low-attenuation materials.

POM standard rods are materials with higher density and larger refractive index gradients, and their phase gradient diagrams show obvious edge mutation characteristics in Figure 3f, which further verifies the edge resolution ability of the method when dealing with high refractive index boundaries.

Based on the imaging results of the three sample groups, the proposed method demonstrates excellent phase sensitivity and edge fidelity when dealing with materials exhibiting significant density variations or weak absorption. It shows strong adaptability and reliable capability for structural identification.

**Figure 4:**
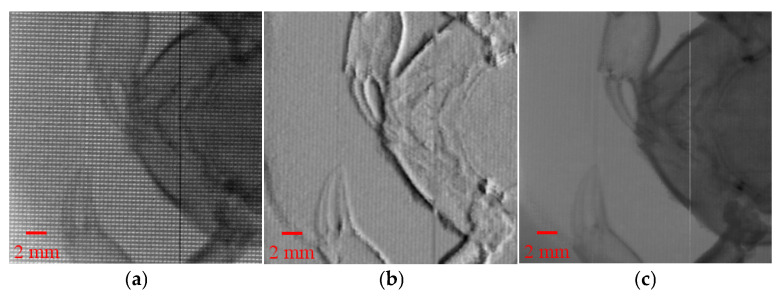
Phase gradient imaging results of a biological sample (crab). (**a**) Raw modulation image; (**b**) Reconstructed phase gradient image showing clear edges and internal structural details; (**c**) Absorption image, provided for comparison with the phase gradient image.

To further evaluate the imaging capability of the proposed method on complex biological samples, a crab was selected for testing. The experimental results are shown in Figure 4, where Figure 4a displays the raw modulation image and Figure 4b shows the reconstructed phase gradient image. Figure 4c shows the absorption image. Here, the distance from the light source to the hole array modulator is 50 cm, and the distance from the hole array to the detector is 25 cm. In the phase gradient image, the edges and some internal details of the crab sample can be clearly observed, especially in the crab claw region, where the effect presented by phase contrast imaging is significantly better than that of absorption imaging.

This indicates that the method can effectively extract phase information from weakly absorbing biological samples, overcoming the limitations of traditional absorption imaging in terms of insufficient contrast in low-density biological tissues.

We also conducted additional experiments to examine the effect of changes in geometric conditions on microbeam contrast and phase gradient reconstruction quality by varying the source-mask distance(z_SM_) and the mask-detector distance(z_MD_). According to the formula:

$$b=s\cdot \frac{z_{MD}}{z_{SM}},$$

where b is the spot size on the detector, and s is the focal size.

**Figure 5:**
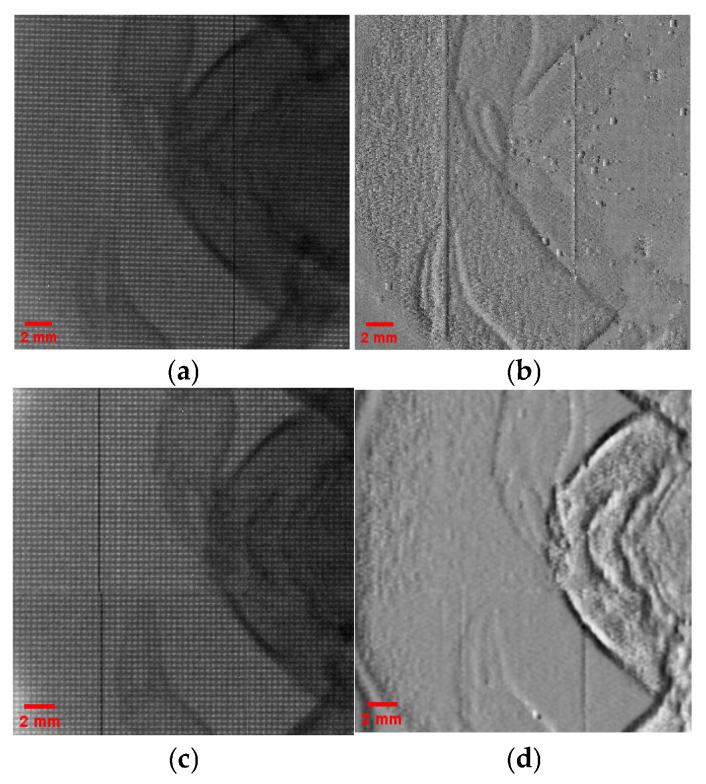
Experimental results under different geometric conditions. (**a**,**b**) are the original image and phase gradient reconstruction of one set of experiments, respectively; (**c**,**d**) are the original image and phase gradient reconstruction of another set of experimental data.

The experimental results are shown in Figure 5. Figure 5a,b are the raw image and phase gradient map of one set of experimental data, respectively. Under this condition, the size of each microbeam on the detector is approximately 240 μm. Compared to the size of microbeam about 200 μm in Figure 4, it can be seen that as the ratio z_MD_/z_SM_ increases, the spot size on the detector gradually increases, and overlap occurs between adjacent spots, leading to a reduction in microbeam contrast and a decline in the quality of phase gradient reconstruction. Figure 5c,d are the raw image and phase gradient map of another set of experimental data, where the size of each microbeam on the detector is about 160 μm, indicating a decrease in spot size. Due to the reduced number of pixels per microbeam used for calculations, the accuracy of the results is affected, and the quality of phase gradient reconstruction also decreases. These results suggest that excessive expansion or undersampling of microbeams can reduce the reliability of phase gradient reconstruction.

In summary, the experimental results demonstrate that the proposed beam tracking imaging method is not only suitable for samples such as pencils, PMMA rods, and POM rods, but also well-adapted for structural imaging of weakly absorbing and biological samples. The method has a certain potential for visual identification of biological structures and low-density composite materials.

## 4. Conclusions

This study proposes a simple X-ray phase-contrast imaging system based on the beam tracking method. By introducing a periodic absorption pinhole array between the X-ray source and the sample, the radiation field is divided into multiple micro-beams. The displacements of these micro-beams caused by the sample are then used to extract absorption and phase gradient information from the recorded images. Compared with conventional phase-contrast imaging techniques, this method offers a simpler system configuration, lower requirements for source coherence and detector resolution.

Although the method benefits from structural simplicity, it currently relies on two-dimensional step-scanning to acquire multiple images for full-field signal reconstruction, which limits its real-time performance and imaging efficiency to a certain extent. Moreover, the acquisition of large datasets increases system runtime and storage demands, making it less favorable for rapid detection and online applications. In addition, since the displacement of micro-beams on the detector is typically small, the method still requires a sufficient signal-to-noise ratio and detector resolution, which may impact the reconstruction quality of weak phase signals.

However, these limitations are expected to be overcome in the future. In subsequent research, we will explore methods to reduce the number of steps, and even adopt a single exposure method, which allows for the simultaneous extraction of absorption and phase information from a single image without step scanning, in order to improve imaging speed and overall efficiency.

Overall, the beam tracking X-ray phase contrast imaging method proposed in this study can simultaneously extract phase gradient information and absorption information under large focal spot and conventional detection conditions. This method significantly reduces dependence on high-coherence light sources and high-resolution detectors, laying a foundation for its promotion in practical applications.

## Data Availability

The data presented in this study are available on request from the corresponding author.

## Abbreviations

The following abbreviations are used in this manuscript:

BT	Beam tracking
PMMA	polymethyl methacrylate
POM	polyoxymethylene
